# UK survey of occupational therapist’s and physiotherapist’s experiences and attitudes towards hip replacement precautions and equipment

**DOI:** 10.1186/s12891-016-1092-x

**Published:** 2016-05-25

**Authors:** T. O. Smith, C. M. Sackley

**Affiliations:** School of Health Sciences, University of East Anglia, Queen’s Building, School of Health Sciences, University of East Anglia, Norwich Research Park, Norwich, NR4 7TJ UK; Division of Health and Social Care Research, King’s College, London, UK

**Keywords:** Total hip replacement, Equipment, Movement restriction, Dislocation, Rehabilitation

## Abstract

**Background:**

Total hip replacement (THR) is one of the most common orthopaedic procedures in the United Kingdom (UK). Historically, people following THR have been provided with hip precautions and equipment such as: raised toilet seats and furniture rises, in order to reduce the risks of dislocation post-operation. The purpose of this study was to determine current practices in the provision of these interventions in the UK for people following primary THR.

**Methods:**

A 27-question, self-administered online survey was developed and distributed to UK physiotherapists and occupational therapists involved in the management of people following primary THR (target respondents). The survey included questions regarding the current practices in the provision of equipment and hip precautions for THR patients, and physiotherapist’s and occupational therapist’s attitudes towards these practices. The survey was disseminated through print and web-based/social media channels.

**Results:**

170 health professionals (87 physiotherapists and 83 occupational therapists), responded to the survey. Commonly prescribed equipment in respondent’s health trusts were raised toilet seats (95 %), toilet frames and rails (88 %), furniture raises (79 %), helping hands/grabbers (77 %), perching stools (75 %) and long-handled shoe horns (75 %). Hip precautions were routinely prescribed by 97 % of respondents. Hip precautions were most frequently taught in a pre-operative group (52 % of respondents). Similarly equipment was most frequently provided pre-operatively (61 % respondents), and most commonly by occupational therapists (74 % respondents). There was variability in the advice provided on the duration of hip precautions and equipment from up to 6 weeks post-operatively to life-time usage.

**Conclusions:**

Current practice on hip precautions and provision of equipment is not full representative of clinician’s perceptions of best care after THR. Future research is warranted to determine whether and to whom hip precautions and equipment should be prescribed post-THR as opposed to the current ‘blanket’ provision of equipment and movement restriction provided in UK practice.

**Electronic supplementary material:**

The online version of this article (doi:10.1186/s12891-016-1092-x) contains supplementary material, which is available to authorized users.

## Background

Approximately 68,845 total hip replacements (THR) were performed in the NHS in England and Wales in 2014 [1]. Although the majority of patients who undergo this procedure are elderly, aged 65 years or above [2], younger patients in their 30s or 40s may also receive THR, particularly for conditions such as ankylosing spondylitis, rheumatoid arthritis, or avascular necrosis of the femoral head secondary to trauma [3, 4]. A THR allows these patients to return to their normal tasks of daily living and recreational activities, where their previous hip pain and weakness is replaced with a pain-free and reliable hip [5].

Total hip replacement dislocation occurs in 3 % to 19 % of primary THRs [6]. It is the second most common complication after aseptic loosening, and it represents a physical and mental disabling for the patient [6–8]. The aetiology of THR dislocation is multi-factorial. It includes surgical factors such as component mal-positioning, soft tissue laxity, and component or anatomical impingement [9]. Patient’s previous medical conditions such as: neuromuscular and cognitive disorders, psychosis and alcoholism also influence the risk towards developing hip dislocation after surgery [10, 11]. The risk of dislocation is acknowledged to be greatest after 3 months post-operatively, and all patients are instructed into learning standard hip precautions [7, 12, 13]. As a result, historically, patients have been taught standard hip precautions [11, 14]. These include: avoiding hip flexion beyond 90°, adduction beyond the mid-line and internal and external rotation greater than 20° [15]. Equipment such as raised toilet seats, long-handled reaching devices, perching stools and chair raises have been provided to prevent patients moving into these positions. Patients have also been advised not to sleep on the side of their THR. These were originally aimed to avoid injuries and to aid the soft tissue repair after surgery.

Hip precautions and equipment have been a major cause of discontent for patients, as it slows down their return to daily activities [16]. Moreover, some studies show that this may potentially slow the rehabilitation process since physical activity and exercise are regarded as essential elements in the rehabilitation of this population [17]. Furthermore precautions and equipment may be associated with a substantial economic and environmental burden in the provision, returning and cleaning of equipment such as abduction pillows, raised toilet seats or chair frames [18].

Recent studies have investigated the clinical importance of hip precautions on dislocation rates. Four studies have assessed the clinical outcomes of removing hip precautions and restrictions on outcomes [16, 19–21]. They reported that not providing THR precautions had no effect on the dislocation rates, whilst allowing greater early functional outcomes compared to teaching precautions following primary THR (*p* < 0.05). Dislocation rates ranged from 0 % to 0.6 % lower than previously reported in a consensus of 1 % dislocation rate for those prescribed hip precautions and equipment.

Whilst these findings have questioned the use of hip precautions and equipment, there remains widespread use of this advice and these devices [22]. A previous survey of UK occupational therapy provision following THR reported national uncertainty regarding the justification for hip precautions, the correct timescales in which it would be useful to follow them, and the amount of time spent while teaching patients about them [22]. Whilst this previous survey provides valuable data on equipment provision, it was directed towards occupational therapists. The purpose of this survey was therefore to address this and to include physiotherapist’s attitudes toward equipment provision and precaution advice. Therefore the aim of this study was to evaluate the overarching question: what are current practices and attitudes of UK occupational therapists and physiotherapists in the provision of equipment and hip precaution advice to people who receive a THR?

## Methods

**Fig. 1:**
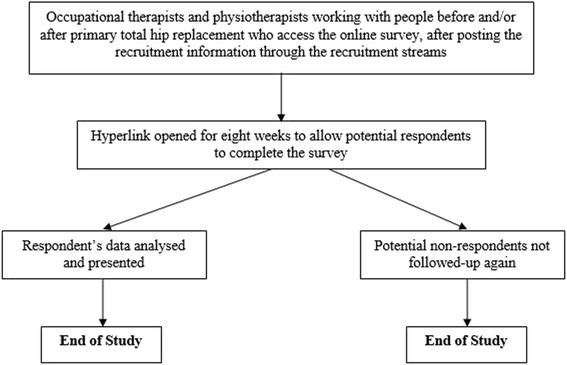
Study flow diagram

An electronic survey was undertaken to answer the research questions. The study process is illustrated in Fig. 1.

### Survey

A self-administered online survey (through the SurveyMonkey (www.surveymonkey.com) platform) consisting of 27 questions was developed (Additional file 1). This survey was structured to answer the following research questions: (1) what are the current practices of occupational therapists and physiotherapists on the provision of post-operative equipment and hip precaution advice following primary total hip replacement; (2) what are the attitudes of occupational therapists and physiotherapists to these practices; (3) are there any patient groups where these practices are modified or amended due to differences in circumstances or patient characteristics; and (4) what are the attitudes of occupational therapists and physiotherapists towards the conduct of research to assess the use of post-operative equipment and hip precaution advice following primary total hip replacement? The survey provided partial closed-ended questions, requiring a categorical response.

### Participants and recruitment

The survey was advertised to occupational therapists and physiotherapists working in the UK who treat patients before and/or after primary THR. We did not exclude respondents based on level of experience, professional grade, or location and type of hospital worked within (i.e. general hospital or specialist orthopaedic centre). The survey was disseminated through print channels (Frontline and the OT News which all members of the Chartered Society of Physiotherapy and College of Occupational Therapy in the UK receive), and electronically through iCSP, Frontline and OT News websites and four Twitter accounts managed by the research team. The survey was open for a total of 8 weeks, with fortnightly reminders posted through print and electronic recruitment streams to prompt potential respondents.

### Data analysis

Descriptive statistics and frequency distributions were used to collectively assess all completed surveys. The data was presented as frequency distributions and mean values with standard deviations where appropriate. When respondents had the choice of providing more than one response for a specific question, the percentage of responses to that specific question’s response option was calculated to reflect all responses rather than taking a single response per individual. For open-question responses, the frequency of practices and attitudes were recorded. Data analysis was undertaken on Statistical Package for the Social Sciences (SPSS) version 18.0 (SPSS Inc, Chicago, Illinois).

## Results

### Respondents

A total of 170 individuals responded and completed the survey. This consisted of 87 physiotherapists (51) and 83 occupational therapists (49 %), representing 170 different health trusts.

### Provision of equipment

The results relating to the provision of equipment are presented in Table 1.

**Table 1:** Data on responses related to equipment provision for people who receive primary THR

	% (Frequency)
Timing of provision of equipment	
Pre-operatively	61 (104)
Post-operative during an in-patient stay	27 (46)
Post-operatively after an in-patient stay	8 (14)
Did not respond	4 (6)
Professional who prescribed equipment	
Occupational therapists (or Occupational Therapy assistant)	74 (126)
Physiotherapists (or Physiotherapy assistant)	11 (18)
Generic therapy advanced practitioners	14 (24)
Nurses	0.5 (1)
Did not respond	0.5 (1)
Who delivers and fits equipment	
Occupational Therapist (or Occupational Therapy Assistant)	41 (69)
Occupational Therapy Technician	22 (38)
Physiotherapist (or Physiotherapy Assistant)	6 (11)
Generic therapy advanced practitioner	5 (8)
Social services	10 (17)
External organisation/company (e.g. Nottingham Rehabilitation Supplies)	50 (85)
Patient/Family/Carer	8 (14)
Joint social services/NHS service	1 (1)
Did not respond	0
Specific groups who would not routinely receive equipment	
No specific exceptions	76 (130)
Cognitively impaired	2 (4)
The younger patient (aged 60 or below)	1 (1)
Very ‘fit’ patients (e.g. runners or those who engage in vigorous physical activity)	3 (5)
Patients from residential or nursing homes	2 (3)
Did not respond	16 (27)
Specific groups who would definitely receive equipment	
No specific exceptions	59 (101)
Cognitively impaired	6 (10)
The younger patient (aged 60 or below)	7 (12)
The older patient (age 85 years or over)	11 (18)
Very ‘fit’ patients (e.g. runners or those who engage in vigorous physical activity)	6 (11)
Patients who had a functional limitation (i.e. unable to functionally raise from a toilet or chair)	24 (40)
Posterior surgical approach	1 (2)
Did not respond	0
Factors which dictate clinical reasoning	
Surgeon protocol/care pathway	58 (99)
Therapy protocol/care pathway	46 (79)
Surgical approach (i.e. posterior, anterior, anterolateral)	18 (31)
My clinical assessment of the patient	41 (70)
Research and evidence-based guidelines	18 (30)
Did not respond	0

*NHS* National Health Service, *THR* total hip replacement

**Fig. 2:**
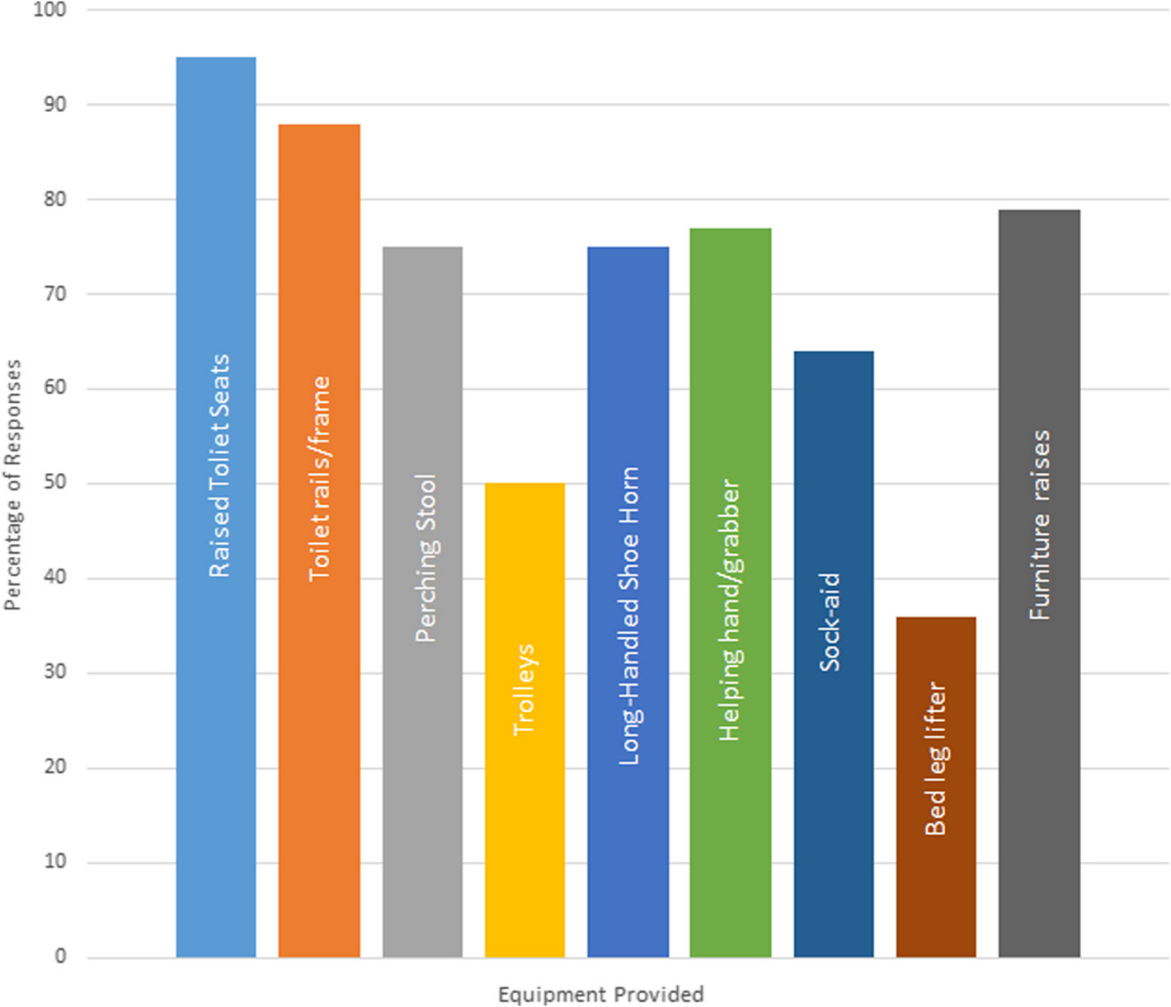
Bar chart presenting the frequency of responses relating to the type of equipment provided to people following THR

It was routine practice to provide equipment to people who undergo primary THR in 87 % of trusts (*n* = 148). However only 54 % of respondents felt that this group of patients should routinely receive such equipment. The frequency of different types of equipment used by respondents is presented in Fig. 2. The most commonly prescribed pieces of equipment were raised toilet seats (95), toilet frames and rails (88), furniture raises (79), helping hands/grabbers (77), perching stools (75) and long-handled shoe horns (75 %). In the majority of cases, this was provided pre-operatively (61 %), and most commonly provided by occupational therapists (74 %). Equipment was provided by physiotherapists in 11 % of trusts. Currently, equipment is fitted in people’s homes by external organisations (such as Nottingham Rehabilitation Supplies; (50 %) or by occupational therapist or occupational therapy technicians (63 %) (Table 1).

**Fig. 3:**
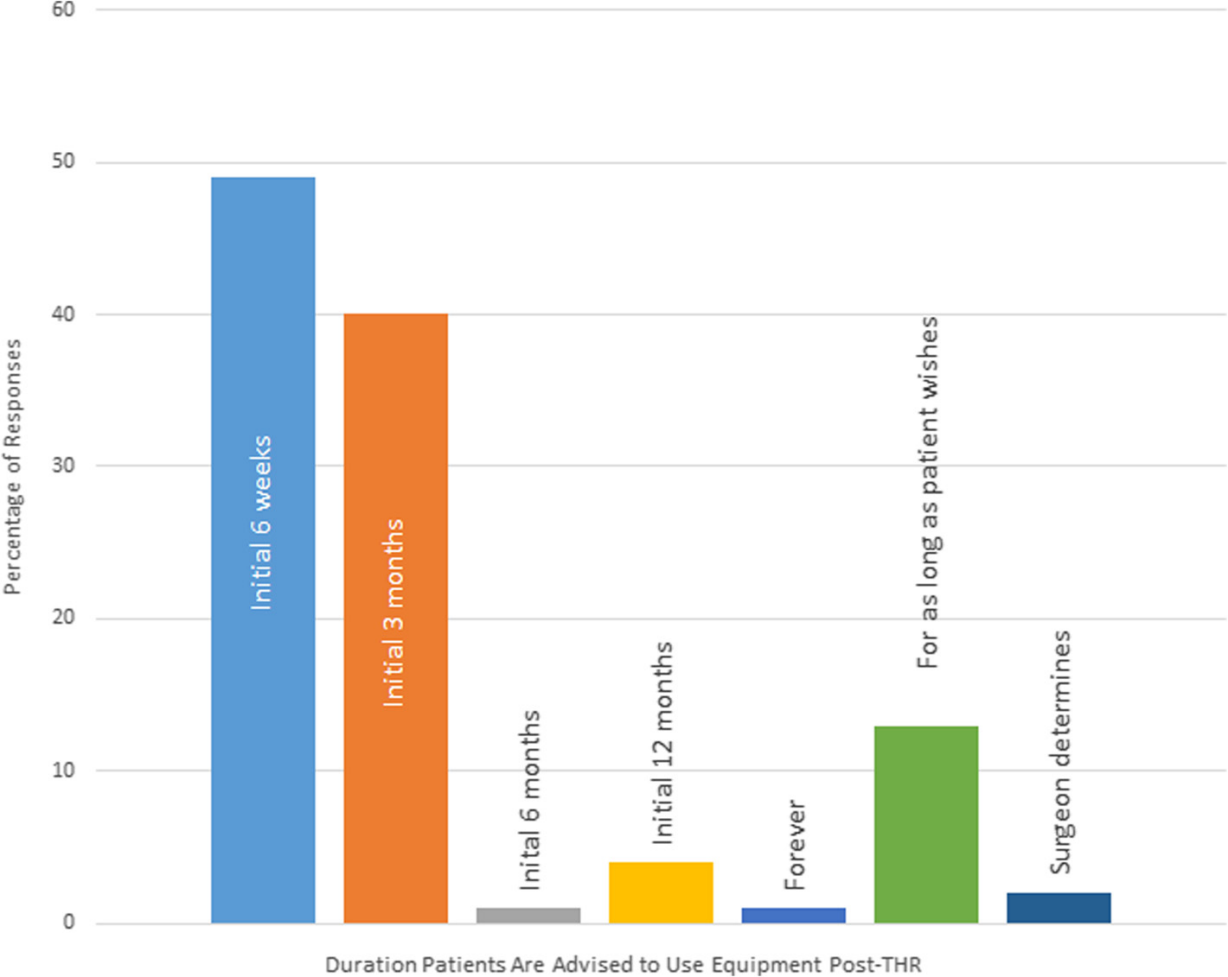
Bar chart presenting the frequency of responses relating to the duration of time recommended that equipment should be used by people following THR

There is national variability in the duration to which patients are currently advised to use their provided equipment post-THR (Fig. 3). The majority of Trusts recommend patients use such equipment for between 6 weeks (49) to 3 months (40), whilst 13 recommend patients use the equipment for as long as they feel appropriate, and 4 % recommend that equipment is used for the first 12 post-operative months. Seventy-six percent of respondents reported that all their patients received equipment following primary THR. Three percent of respondents suggested that they would not routinely provide equipment to very ‘fit’ patients such as runners or those who engage in vigorous physical activity. Two percent did not routinely provide equipment to people with cognitive impairment. Six percent of respondents also reported that they would definitely prescribe equipment to this group of people. When asked who would definitely receive equipment, subgroups identified by respondents included younger patients (7), patients aged 85 years or over (11), very fit patients (as defined above; 6) and those who had a functional limitation (24 %).

There was considerable overlap on which factors determined clinical reasoning for the prescription of equipment. This was most frequently based on multiple reasons including: surgical protocol (58 %), therapy protocol (46 %) or the clinician’s personal assessment (41 %).

### Provision of Hip precaution (movement) advice

The results relating to the provision of advice on hip precautions and movement restrictions are presented in Table 2.

**Table 2:** Data on responses related to hip precautions and movement restriction for people who receive primary THR

	% (Frequency)
Duration patients advised to follow hip precautions	
For the first 6 weeks post-operation.	55 (94)
For the first 3 months post-operation.	36 (62)
For the first 12 months post-operation.	2 (3)
Forever after their total hip replacement.	4 (6)
For as long as the patient feels appropriate.	4 (7)
Did not respond	0
Principle providers of hip precaution advice and information	
Occupational Therapists (or Occupational Therapy Assistants)	75 (128)
Physiotherapists (or Physiotherapy Assistants)	71 (121)
Generic therapy advanced practitioners	15 (36)
Nurses (and Health Care Assistants)	27 (46)
Surgeons and medical team	30 (51)
Did not respond	0
Timing of provision on hip precaution advice and information	
Pre-operatively in a one-on-one consultation	39 (67)
Pre-operatively in a group setting with other THR patients	52 (89)
Pre-operative in the form of a leaflet or DVD or website	29 (50)
Post-operatively during the patient’s in-patient stay	43 (73)
Post-operative after hospital discharge	10 (17)
Did not respond	0
Specific groups who would not routinely receive hip precaution advice	
No specific exceptions	79 (134)
Cognitively impaired	7 (12)
The younger patient (aged 60 or below)	2 (4)
The older patient (age 85 years or over)	1 (1)
Very ‘fit’ patients (e.g. runners or those who engage in vigorous physical activity)	1 (2)
Anterior surgical approach	2 (3)
Constrain hip replacement	1 (1)
Did not respond	8 (13)
Specific groups who would definitely routinely receive hip precaution advice	
No specific exceptions	74 (125)
Cognitively impaired	5 (8)
The younger patient (aged 60 or below)	6 (11)
The older patient (age 85 years or over)	9 (15)
Very ‘fit’ patients (e.g. runners or those who engage in vigorous physical activity)	9 (16)
Posterior surgical approach	1 (2)
Did not respond	0
Factors which dictate clinical reasoning	
Surgeon protocol/care pathway	72 (122)
Therapy protocol/care pathway	50 (85)
Surgical approach (i.e. posterior, anterior, anterolateral)	25 (42)
My clinical assessment of the patient	44 (74)
Research and evidence-based guidelines	28 (47)
Did not respond	0

*THR* total hip replacement

**Fig. 4:**
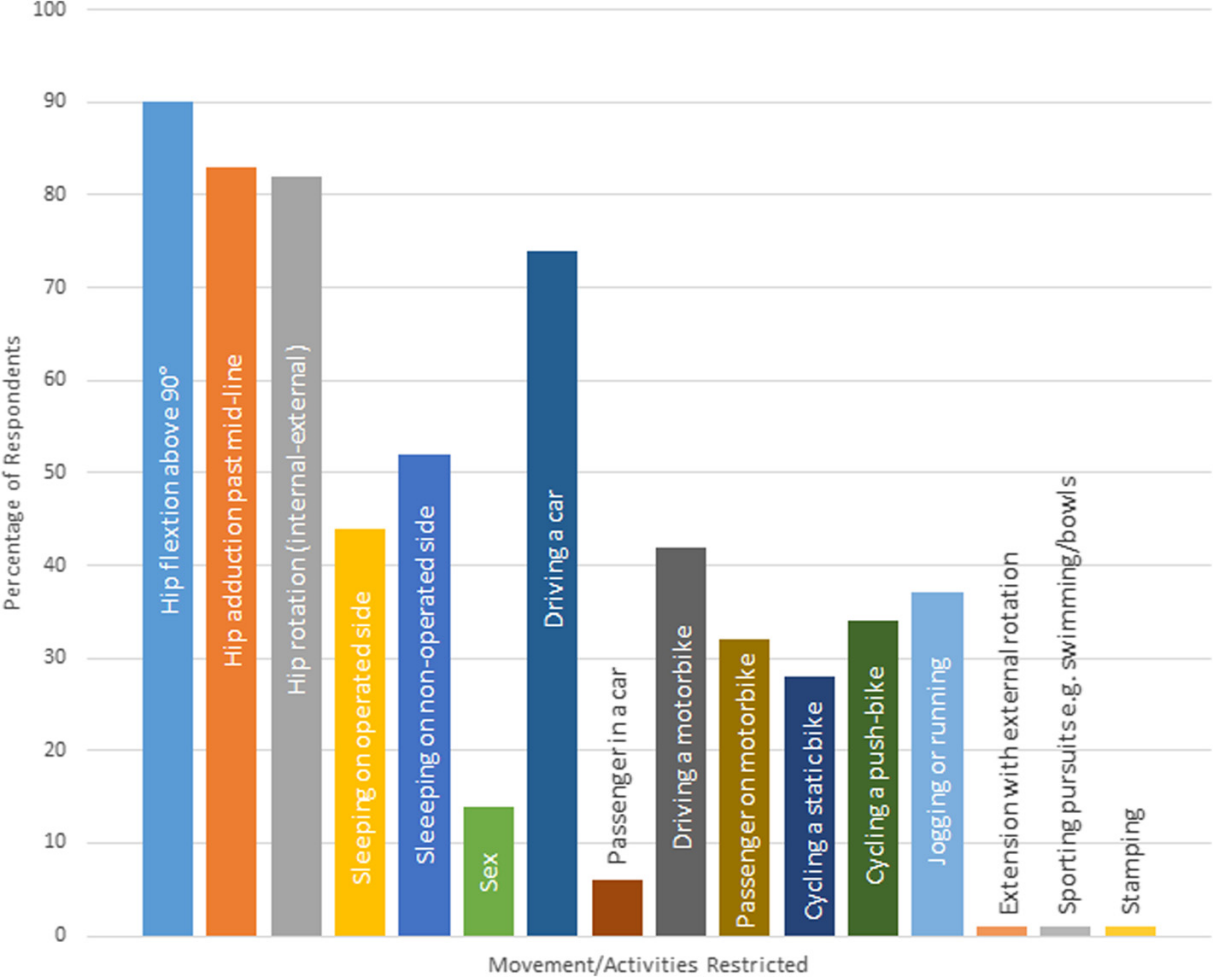
Bar chart presenting the frequency of responses relating to the type of movement restriction advised to people following THR

Ninety-seven percent of respondents reported that they routinely provided advice on hip precautions. However 25 % of participants felt that this should not be a routine practice. Figure 4 presents the frequency of each different movement and activity restrictions which are advised to patients following THR. Movements which were most commonly advised to avoid included hip flexion (90), adduction (83), and rotation (82 %), whilst specific activities instructed to be avoided included driving a car (74 %), sleeping on the non-operated side (52 %), sleeping on the operated side (44 %) and driving a motorbike or scooter (42 %). The continuation of these varied from the first six post-operative weeks (55 %), to up to 3 months (36 %), to the first 12 months (2 %). Four percent of respondents reported instructing their patients to continue with these precautions indefinitely.

Advice on hip precaution and movement restriction is provided by a variety of clinicians. Seventy-five percent reported that occupational therapists provide this information, 71 % reported that physiotherapists provided this information, whilst 30 and 27 % of respondents reported that surgical teams and nursing staff provide this information to their patients as well. Similarly this information is provided through a variety of streams. Principally, people are informed about hip precautions in a pre-operative group (52 %). This information was also provided post-operatively during the patient’s in-patient stay either face-to-face (43 %) or using a leaflet, DVD or website (29 %). Ten percent of respondents reported that their patients are provided with this information post-operatively after hospital discharge.

Seventy-nine percent of respondents reported that all patients were advised on hip precautions or movement limitation, with few exceptions. Seven percent reported that they would not routinely provided this advice for people with cognitive impairment, and 2 % reported not providing such information routinely to those who had undergone an anterior surgical approach. Seventy-four percent of respondents reported that they had no specific exceptions on who would definitively receive post-operative advice on movement restriction. Of those who did have exceptions, five percent reported definitely providing advice for those with cognitive impairment, six percent provided this for younger patients (aged 60 or below), and nine percent reported definitely providing this for older patients (aged 85 years or older). Nine percent reported definitely providing this for people who were considered ‘very fit’ i.e. participated in vigorous exercise. There was overlap on clinical reasoning underpinning this decision. Factors which informed clinical decision making for whether or not to provide advice on movement limitation was most commonly the surgical protocol (76 %), therapy protocol (5 %) but also clinical assessment of the patient reported in 44 %.

### Clinician’s perceptions

When asked whether respondents would like to change their current practice, 48 % reported that they would if they could. These changes were largely around decision-making on the provision of equipment and movement restriction on an individual basis (86 %). Eleven percent of individuals reported they would remove the provision of both equipment and precautions for people post-THR. Sixty-six percent of respondents felt that there needed to be more research on the provision of equipment, with 88 % recommending more research on the provision of hip precautions following primary THR.

## Discussion

The findings of this survey indicate that whilst equipment and hip precautions are routinely provided for the majority of people following primary THR, this is largely questioned by UK occupational therapists and physiotherapists. This survey has reinforced that these practices are widespread, particularly in occupational therapy practice, but are not necessarily evidence-based given the previous evidence in relation to dislocation events [16, 19–21]. Respondents have acknowledged a need for further research to determine which specific subgroups of patients, such as those who have a functional limitation and need equipment to facilitate independence be undertaken rather than the continuation of the current ‘blanket’ provision of equipment and movement restriction provided in the UK.

This survey’s conclusions are largely in agreement with Drummond et al’s [22] earlier survey of occupational therapy practice following hip replacement. This provides further evidence that clear uncertainty exists nationwide regarding hip precautions after THR in both occupational therapy practice in Drummond et al’s survey [22] and physiotherapists as well as occupational therapists in this survey. The variability in national responses previously seen in this survey is mirrored in our findings. For instance, there is considerable national discrepancy in the duration that equipment and movement restriction are advised for patients, with patients advised to use the equipment during a 6 week period, or it can be until the patient or orthopaedic surgeon feels appropriate. Similarly there is variability as to which specific groups of patients should or should not receive these interventions. This may be attributed to a poor evidence-base in this field, allowing considerable uncertainty on these key parameters. Until a more robust and well-disseminated evidence-base is made, on which national clinical guidelines can be made to inform surgical or therapy protocols in individual trusts, such variability will be evident.

A number of respondents reported basing their clinical recommendations on whether patients under-went an anterior or posterior surgical approach. Whilst anatomically an anterior surgical approach may place people at greater risk of an anterior dislocation during hip extension and rotation movements, as opposed to a posterior approach which may increase the risk of dislocation during hip flexion and rotation, the evidence remains inconclusive [23]. Nonetheless, this theoretical approach may warrant further study, particularly given the variability in surgical approach used in clinical practice [1].

There is also considerable uncertainty as to who could most benefit from hip precautions and equipment. As Tables 1 and 2 demonstrate, there was inconsistency as to whether people with cognitive impairment, or people regarded as ‘very fit’ should or should not be highlighted for these interventions due to their perceived greater risk of an adverse event. This inconsistency may be attributed to a lack of research in the field to support clinical decision-making. The only consistent finding was that hip equipment should be provided to people who have a functional limitation. Therefore in this instance, equipment is used as an intervention for functional inability rather than to reduce hip dislocation risks which may be perceived as a different perceptive on the prescription of these interventions.

We attempted to investigate the variability in duration in which patients are advised to use equipment or hip precautions. It was hypothesised that this may be related to surgeon instruction rather than variation in occupational therapy or physiotherapy practice. As demonstrated by this survey’s findings, surgeon pathway or protocol dictated clinical reasoning for 72 and 58 % of respondents in respect to movement restriction and equipment provision. When further analysed, there appeared no clear difference between those clinicians who reported different clinical reasoning (i.e. therapy protocol or clinicians assessment) compared to surgeon protocol for the duration or movement restriction or equipment provision. This may have been due to the relatively small number of responses for these alternative clinical reasoning approaches. Accordingly further investigation is warranted to better understand what factors inform rehabilitation and recovery pathways for people following THR. This would have a significant benefit when considering how to implement multidisciplinary rehabilitation changes for this setting.

There is considerable support for future research in this area amongst respondents. The identification of who could most benefit from the use or withdrawal of equipment and movement restriction is paramount. A current Cochrane review being undertaken by the research team which suggests that functional outcomes, including return to activities of daily living, gait progression and return to driving a car, may be higher for people who are not prescribed equipment or post-operative advice [24]. This is re-iterated in Barnsley et al’s [25] review which concluded that hip precautions may slower return to activities, decrease patient satisfaction, have significant expense, whilst no reducing the rate of THR dislocation. Therefore clinical outcomes may be superior through the withdrawal rather than addition of these interventions. This may provide a further motivation for occupational therapists, physiotherapists, nurses and surgeons to re-evaluate their shared knowledge on the use of these interventions and to consider, as future research develops, whether these intervention are still valuable for these patients in the 21^st^ century.

Whilst the findings of this survey are based on the responses of physiotherapists and occupational therapists in the United Kingdom, the literature would suggest that these trends may reflect the clinical practices from other countries, particularly the United States of America (USA). Studies such as Schmidt-Braekling et al. [26], Restrepo et al. [16] and Ververeli et al [21] from the USA have previous questioned the current value of the implementation of ‘blanket’ hip precautions for people following THR. However no national survey data is available assessing hip precaution or equipment provision for people following THR in the USA. Accordingly, further studies are needed in order to explore how the responses from our survey reflect practices in other healthcare services such as Europe, the USA, Asia and Australia. Through this, it will be possible to better understand how hip precautions and post-operative equipment are employed for a THR who may have different physical demands and perceptions of post-operative recovery compared to the UK and other populations. Finally, it was not the objective of this survey to explore the effect of level of experience or prevailing surgical approach (i.e. anterior-posterior) on equipment or hip precaution provision. Whilst the data provided some indication on a difference in practice based on the latter, further, more in-depth investigation using qualitative research methods may be a valuable area for future study. This may be particularly important to explore whether the level of experience and personal attitudes of occupational therapists and physiotherapists impact on their prescription of equipment or hip precautions following THR.

## Conclusions

Following primary THR, people are commonly prescribed hip precautions and equipment, mostly during the indicial 3 months post-operatively, which can restrict function. Whilst this survey has indicated that this is a nationwide practice, UK occupational therapists and physiotherapists currently question whether this blanket approach to providing these interventions is justified, or whether certain individuals should or should not receive these treatments. Further research is warranted to further explore the adoption of these interventions and to determine who could benefit the most from the addition or removal of hip equipment or movement restrictions following primary THR.

### Data

All relevant data is included in the manuscript and the supporting information files.

## Abbreviations

DVD, Digital video disc; NHS, National Health Service; THR, Total hip replacement; UK, United Kingdom; USA, United States of America

## Additional file

Additional file 1: Survey. (XLS 205 kb)

## References

[1] National Joint Register. StatsOnline. http://www.njrcentre.org.uk/njrcentre/Healthcareproviders/Accessingthedata/StatsOnline/NJRStatsOnline/tabid/179/Default.aspx (2015, Accessed 19 Aug 2015).

[2] Lohmander LS, Engesaeter LB, Herberts P (2006). Standardized incidence rates of total hip replacement for primary hip osteoarthritis in the 5 Nordic countries: similarities and differences. Acta Orthop.

[3] Waddell JP, Morton J, Schemitsch EH (2004). The role of total hip replacement in intertrochanteric fractures of the femur. Clin Orthop Relat Res.

[4] Sorbie C (2003). Arthroplasty in the treatment of subcapital hip fracture. Orthopedics.

[5] Jones CA, Voaklander DC, Johnston DW (2000). Health related quality of life outcomes after total hip and knee arthroplasties in a community based population. J Rheumatol.

[6] Bourne RB, Mehin R (2004). The dislocating hip: what to do, what to do. J Arthroplasty.

[7] Woo RY, Morrey BF (1982). Dislocations after total hip arthroplasty. J Bone Joint Surg Am.

[8] Dorr LD, Wan Z (1998). Causes of and treatment protocol for instability of total hip replacement. Clin Orthop Relat Res.

[9] Patel PD, Potts A, Froimson MI (2007). The dislocating hip arthroplasty: prevention and treatment. J Arthroplasty.

[10] Soong M, Rubash HE, Macaulay W (2004). Dislocation after total hip arthroplasty. J Am Acad Orthop Surg.

[11] Mahoney CR, Pellicci PM (2003). Complications in primary total hip arthroplasty: avoidance and management of dislocations. Instr Course Lect.

[12] Khatod M, Barber T, Paxton E (2006). An analysis of the risk of hip dislocation with a contemporary total joint registry. Clin Orthop Relat Res.

[13] Phillips CB, Barrett JA, Losina E (2003). Incidence rates of dislocation, pulmonary embolism, and deep infection during the first six months after elective total hip replacement. J Bone Joint Surg Am.

[14] Berry DJ (2001). Unstable total hip arthroplasty: detailed overview. Instr Course Lect.

[15] Lucas B (2008). Total hip and total knee replacement: postoperative nursing management. Br J Nurs.

[16] Restrepo C, Mortazavi SM, Brothers J (2011). Hip dislocation: are hip precautions necessary in anterior approaches?. Clin Orthop Relat Res.

[17] Minns Lowe CJ, Barker KL, Dewey ME (2009). Effectiveness of physiotherapy exercise following hip arthroplasty for osteoarthritis: a systematic review of clinical trials. BMC Musculoskelet Disord.

[18] NHS. NHS Choices. Occupational Therapy – Accessing Occupational Therapy. http://www.nhs.uk/Conditions/occupational-therapy/Pages/accessing-occupational-therapy.aspx (2013, Accessed 13 Feb 2013).

[19] Peak EL, Parvizi J, Ciminiello M (2005). The role of patient restrictions in reducing the prevalence of early dislocation following total hip arthroplasty. A randomized, prospective study. J Bone Joint Surg Am.

[20] Talbot NJ, Brown JH, Treble NJ (2002). Early dislocation after total hip arthroplasty: are postoperative restrictions necessary?. J Arthroplasty.

[21] Ververeli PA, Lebby EB, Tyler C (2009). Evaluation of reducing postoperative hip precautions in total hip replacement: a randomized prospective study. Orthopedics.

[22] Drummond A, Coole C, Brewin C (2012). Hip precautions following primary total hip replacement: a national survey of current occupational therapy practice. Br J Occupat Therap.

[23] Higgins BT, Barlow DR, Heagerty NE (2015). Anterior vs. posterior approach for total hip arthroplasty, a systematic review and meta-analysis. J Arthroplasty.

[24] Jepson P, Beswick A, Smith TO, et al. Assistive devised, hip precautions, environmental modifications and training to prevent dislocation and improve function after hip arthroplasty (Protocol). Cochrane Database of Systematic Reviews 2013; 11: CD010815. (DOI: 10.1002/14651858.CD010815). 10.1002/14651858.CD010815.pub2 PMC6458012 27374001

[25] Barnsley L, Barnsley L, Page R (2015). Are hip precautions necessary post total hip arthroplasty? A systematic review. Geriatr Orthop Surg Rehabil.

[26] Schmidt-Braekling T, Waldstein W, Akalin E (2015). Minimal invasive posterior total hip arthroplasty: are 6 weeks of hip precautions really necessary?. Arch Orthop Trauma Surg.

